# Assessing the Use of Telepresence-Guided Video-Based Head and Neck Ultrasound Training: A Step towards Minimizing Dependence on Human Resources?

**DOI:** 10.3390/diagnostics13172828

**Published:** 2023-08-31

**Authors:** Philipp Kulas, Bernhard Schick, Johanna Helfrich, Alessandro Bozzato, Dietmar J. Hecker, Lukas Pillong

**Affiliations:** Department for Otorhinolaryngology and Head- and Neck-Surgery, Saarland University Medical Center, Kirrbergerstraße 100, 66421 Homburg, Germany; philipp.kulas@uks.eu (P.K.); bernhard.schick@uks.eu (B.S.); johanna.helfrich@uks.eu (J.H.); alessandro.bozzato@uks.eu (A.B.); dietmar.hecker@uks.eu (D.J.H.)

**Keywords:** head and neck imaging, head and neck ultrasound, video-based teaching, remote teaching

## Abstract

The acquisition of ultrasound skills is an essential part of any medical student’s education. University access restrictions in the context of the COVID-19 pandemic have highlighted the need for digitization in teaching. However, teaching manual skills in online courses has proven to be challenging, not least in terms of human resources. Therefore, the aim of this study was to set up a hybrid head and neck ultrasound course consisting of a preface of video-based self-study followed by supportive instruction by a tutor in telepresence and to evaluate the quality, effectiveness, and feasibility of this teaching method. Thirty-five students were shown video tutorials on systematic ultrasound of the neck course. Learning outcomes were analyzed using self-assessment questionnaires and external assessment by an experienced ultrasonographer. All participants demonstrated statistically significant learning improvement (*p* < 0.001) when comparing self-assessment scores before and after training. The mean self-assessment scores increased from 13.8 to 26.6 for the telepresence-guided group, from 16.6 to 27.3 for the web-based group, and from 14.0 to 26.2 for the in-person group. The external observer assessment also showed improvement, with mean scores of 46.7, 48.1, and 46.5, respectively. Overall results did not significantly differ when comparing different instruction modalities. A telepresence-guided video-based ultrasound course is well suited to teaching ultrasound skills similar to in-person courses and allows a more resource-efficient targeting of student needs.

## 1. Introduction

Sonography is a radiation-free bedside imaging technique that is increasingly relevant in clinical practice due to its cost-effectiveness, availability, and speed. In otorhinolaryngology, it is a valuable tool in differentiating between inflammatory and tumorous lesions of the soft tissues (neck, salivary glands, thyroid gland, and lymph nodes) and remains pertinent in follow-up care. Therefore, ultrasound examination skills are of particular importance.

Ultrasound technology represents a significant expansion of the diagnostic repertoire and must be an integral part of education for future medical physicians [1]. Since the quality, clarity, accuracy, and interpretation of sonographic images largely depend on the examiner’s experience and expertise [2], hands-on teaching has been firmly established as a gold standard in numerous curricula [3,4,5,6,7]. During the COVID-19 pandemic, face-to-face teaching was largely paused, so practical courses could no longer be taught in person due to access restrictions at universities. New teaching concepts were needed to offer practical training to students [8,9,10,11,12].

Establishing and delivering an ultrasound course as part of an undergraduate curriculum is resource-intensive [13,14]. The modern landscape of medical education, marked by challenges such as limited expert availability and growing student numbers, necessitates innovative approaches to optimize human resources while maintaining educational quality. Technology-driven teaching methods offer solutions to these challenges. They enhance efficiency, adapt to diverse learning speeds, ensure standardized content, and facilitate timely updates. By reducing the burden on human resources, institutions can achieve cost savings and provide a more tailored learning experience.

In light of these considerations, a shift towards technology-enhanced learning emerges as a promising avenue, holding the potential to revolutionize medical education by minimizing resource dependency and enriching the academic journey.

Media-enhanced instruction has come into focus as a possibility to conserve human resources. Positive experiences with remote education using augmented reality approaches [15] as well as telemedicine teaching of ultrasound-based procedures have been described [16,17]. Educational programs in general have profited from the use of tutorial videos, particularly when imparting knowledge about practical skills [18,19,20]. We have recently shown that an online-based head and neck ultrasound course (HANUS) led to high acceptance and good student learning success. Nevertheless, the potential and limitations of hybrid learning models need to be further explored to ensure sustainable teaching at university hospitals while still addressing individual student needs.

In this study, our goal was to extend the video-based HANUS course [18] with additional didactic and technical elements to investigate the potential of this method for further training of students with a reduction of human resources. Therefore, we compared the established face-to-face format as well as an online teaching format described in our preliminary work [21] with this hybrid model based on video instruction and concomitant individual remote supervision. The evaluation focused on the quality, effectiveness, and feasibility of the respective course formats.

## 2. Materials and Methods

### 2.1. Study Design and Participant Recruitment

Thirty-five medical students from both pre-clinical and clinical phases of their education (Semesters 4–10) were recruited for this study. These students were selected from ongoing undergraduate classes and were enrolled voluntarily. In accordance with the exploratory nature of our study, participants were selected based on their enrollment in the Human Medicine program at our university. Notably, specific prerequisites related to prior ultrasound proficiency were intentionally omitted as exclusion criteria. This strategic decision was driven by our aim to comprehensively assess the impact of the intervention on individual learning outcomes, allowing us to capture a diverse representation of participants’ baseline skills. In comparison to standard face-to-face sessions for teaching HANUS skills, we designed a hybrid course format that consisted of pre-recorded video instructions followed by live but remote instructions using Microsoft Teams video conferencing tools. As described in our preliminary work [21], the undergraduate ultrasound course was structured as part of the curriculum, which is divided into 45 min units. We, therefore, chose a 90 min timeframe for instruction in this study.

All students provided informed consent to participate in the study. To ensure comparability with previously collected data, we assessed the effectiveness and quality of our course using a pretest–posttest design and a standardized self-assessment questionnaire on a 4-point Likert scale (Table 1). An experienced ultrasound examiner using the Objective Structured Clinical Examination (OSCE) items, as shown in Table 2, conducted a final assessment.

Statistical analysis was performed using GraphPad Prism 7 (GraphPad Software, San Diego, CA, USA). We employed a two-way ANOVA with Sidak’s correction for multiple comparisons to assess the mean differences in the attained self-assessment score values before and after the course. Additionally, we utilized an ANOVA with Tukey’s correction for multiple comparisons to compare the self-assessment score values before and after the course across the various instructional format groups (video-based remote teaching, in-person teaching, and online teaching from our previous work [21]). To compare the score values of ultrasound skills obtained during the OSCE assessment across different teaching formats, the Kruskal–Wallis test with Dunn’s correction for multiple comparisons was used.

### 2.2. Technical Setup

**Figure 1:**
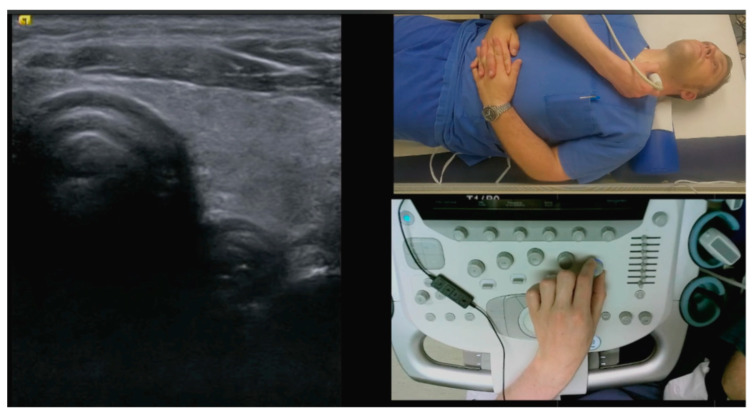
Excerpt from the video guide on how to perform a detailed HANUS examination. The ultrasound transducer- and patient position were displayed in the upper right and the control panel of the ultrasound system in the lower right part of the screen, the corresponding ultrasound image was shown in the left part of the screen. In the audio track of the video, detailed instruction with an explanation of the mapped structures and the handling of the system was given.

A group of 35 students received a 45 min recording set up as a primer on ENT ultrasound skills, prior to the hands-on section of the course. The video sequences were based on curriculum content with a detailed description and explanation of examination steps, device settings, transducer orientation, anatomic landmarks, and systematic examination of the head and neck region. These video sequences were sectioned according to thematic units and edited so that practical execution could be trained following each video unit (Figure 1).

**Figure 2:**
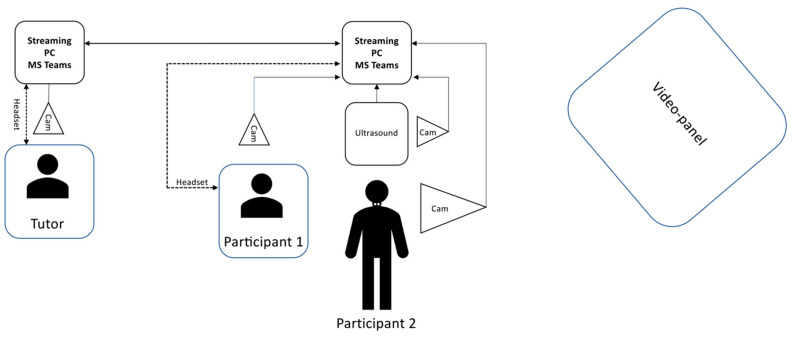
Experimental setup: Illustration of the experimental setup depicting Participant 2 as the examinee and Participant 1 as the examiner. The pre-recorded instructional videos could be viewed by the participants on a large video panel in the examination room. The setup involves the transmission of audio signals, ultrasound images, and a continuous camera feed capturing both participants, facilitated by OBS and Microsoft Teams. The remote tutor, connected via Microsoft Teams, remains virtually present throughout the examination, using a webcam and headset to provide targeted guidance and instructions to both participants from a distance.

This hands-on training was conducted on two ultrasound devices (VINNO 6, VINNO, Suzhou, China, and Siemens Acuson S2000, Siemens Healthcare GmbH, Erlangen, Germany), each of which was integrated into a Microsoft Teams stream with the aid of a virtual camera signal via OBS (Open Broadcaster Software, open source software, version 26.1.0 www.obsproject.com, accessed on 14 December 2020). Additionally, two external cameras (GoProHero9, GoPro, San Mateo, CA, USA) were used to transmit an image of the examination situation, including the patient’s position, the transducer’s position, and the anatomical region being examined. An audio connection was established using a headset, allowing a supervisor physically separated from the students to follow each examination step and interact directly with the students and provide assistance (Figure 2).

Before the start of the course, participants were asked questions regarding their ability to locate anatomical landmarks. Upon completion of the course, another questionnaire was used to assess the individual achievement of learning objectives. Self-assessment scores ranged from 0 to 36 points. Additionally, questions addressed the students’ evaluation of the course format, including didactic quality, comprehensibility of teaching materials, and tutor guidance. After the training, all participants were evaluated by a senior physician of DEGUM (German Society for Ultrasound in Medicine) Level II.

We utilized standardized questionnaires to ensure comparability of the course formats. This enabled us to analyze the data collected in this study and corroborate other data obtained during the current semester on the instructional modalities previously described [21] (in-person and online course). For additional details on the setup of these specific modalities, we refer to our preliminary work.

## 3. Results

To ensure comparability across course formats, we present results from three different instructional modalities: the online format shown in our previous study [21], the face-to-face format with participants recruited during the regular semester, and the newly introduced video-based course format described in this study. Characteristics of the student population are shown in Table 3.

**Figure 3:**
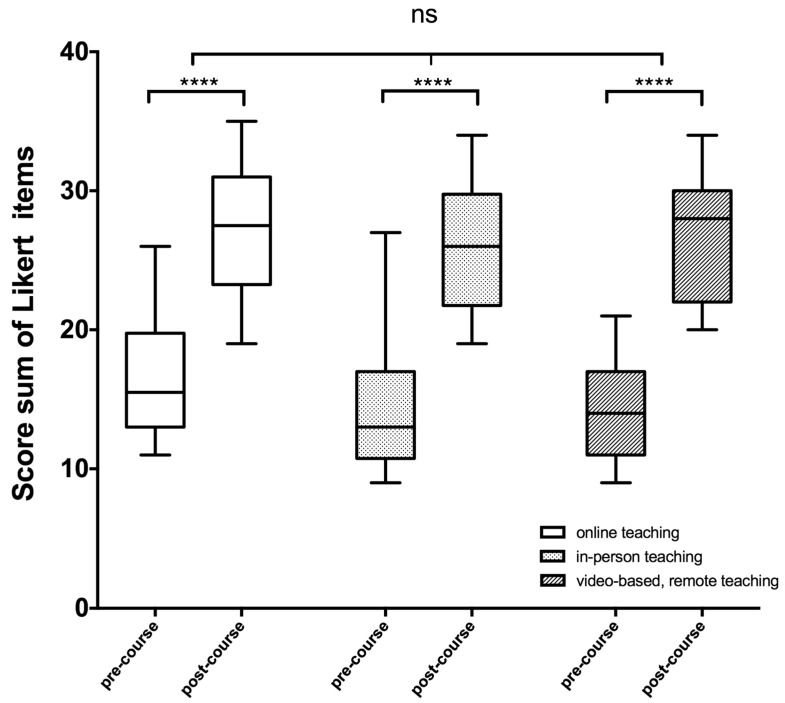
Participants’ self-evaluation of their ultrasound skills before (pre-course) and after (post-course) training. The sum of the 4-point Likert scale score values before and after the course is presented in box plots for each group. The two-way ANOVA revealed no significant (ns = *p* > 0.05) differences among the various course formats, but a significantly higher score after training within each individual group (**** = *p* < 0.0001) compared to the self-assessment prior to the course.

Upon the culmination of any of the three course modalities, all participants demonstrated a notable enhancement in their ultrasound proficiency (Figure 3). Prior to the video-based course with telepresence tutor support, participants had some difficulties locating and identifying anatomical structures such as the submandibular gland during ultrasound examination, with an average score sum of 13.8 on the Likert scale items.

However, after completing the course, most participants in all groups were able to confidently locate and visualize all relevant anatomical structures of the head and neck region, which was reflected in their post-test results (Figure 3).

The group that received video lessons in combination with telepresence tutor support showed a significant improvement (*p* < 0.001) in their self-assessment scores, with a mean score sum of 26.6. In the web-based course, participants improved from a mean score value of 16.6 before the course to 27.3 after the training session. The in-person course participants also showed improvement, with a mean score increase from 14 before to 26.2 after the training session.

**Figure 4:**
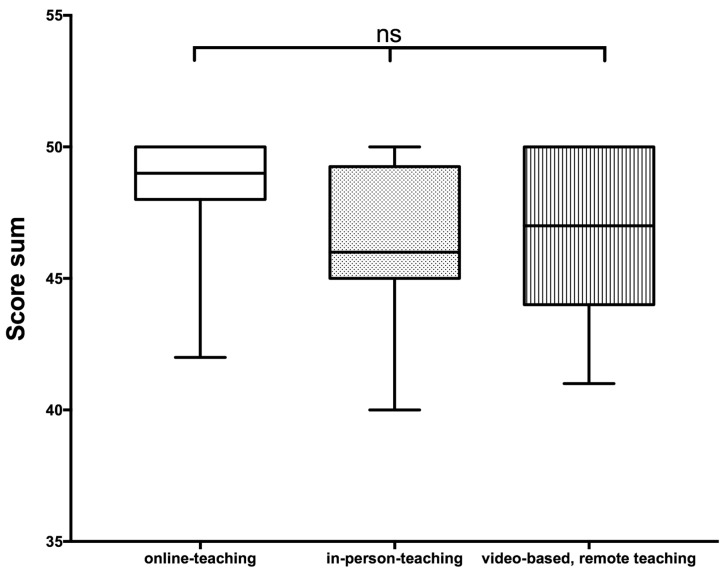
Score values reached in the final evaluation by an experienced US examiner according to the scoring system shown in Table 2. The Kruskal–Wallis test revealed no significant (ns = *p* > 0.05) differences when comparing the attained score values among the various teaching formats.

However, no significant difference in self-assessment scores after course participation was found among the three groups. To provide an objective assessment of the acquired skills, we used the OSCE approach. Following the complete 90 min training session, a senior physician with a DEGUM Level II certification assessed all participants as a second examiner. The video-based course with remote instruction participants had a mean score of 46.7, which was slightly lower but not significantly different from the web-based course participants (48.1) or the in-person class participants (46.5) (Figure 4).

Participants expressed favorable assessments for all three course formats regarding individual learning achievements, time allocation, course organization, and instructional aspects.

Overall, this new teaching format conserves resources. Not only does it require fewer expert staff to manage, but it also puts less of a strain on individual teachers, since the instructional videos can be reused and they were proven to support student learning significantly. The course illustrated that students’ learning processes were markedly improved. During the course of a single session, students changed from a purely passive to an active investigative role.

## 4. Discussion

Firstly, the presented work illustrates that this new ultrasound course concept including multimedia teaching tools was similarly effective as previous designs; secondly, it stimulated students’ learning ability with the need for an active learning attitude; and lastly, it decreased human resources. Application of this concept in a clinical context is not only possible locally but on a far wider scale. Additionally, in the future, the setup can be used to train students over long distances.

Our previous study [21] demonstrated that the virtual transmission of a live patient examination by an instructor represented a viable guideline for students to follow in parallel. The purpose of this study was to expand on our HANUS course to include video tutorials for each separate step of the structured HANUS examination, followed by hands-on sessions with remote feedback and guidance from an ultrasound instructor [21].

A significant advantage of the setup described in our preliminary work [21] that we could also capitalize on for this study was the amount of information that could be transmitted in a single image. The transmission allowed for a double-sided display of the patient and transducer positions, control panel settings, and live ultrasound images. This facilitated direct interaction between the instructor and student. We were able to address issues immediately and directly, and any corrections could be promptly demonstrated.

Another major advantage of our setup was that it allowed us to adapt to an individual learner’s needs and performance. It offered an individually long hands-on time owed to the small group size. Other face-to-face courses offer far less favorable instructor–student or student–ultrasound device ratios, where only one student ever has active examination time on the ultrasound machine, while the rest of the group is less involved [22].

Pre-recorded video tutorials allowed the transfer of condensed packages of information, which students could recall at a time of their choosing. Secondly, it also ensured that all students received equal instruction, based on reliable sources and expert knowledge. Thirdly, incorporating these introductory segments as supported self-study ensured that the hands-on course could be started in medias res. The experience in this study was that students appreciated this new way of teaching markedly. Human resources were conserved since it reduced the number of instructors required while enabling efficient teaching. Our findings are consistent with recent observations by Situ-LaCasse et al., who showed that medical students who have no prior experience with ultrasound can acquire basic hands-on skills in image acquisition after going through online modules with video tutorials [23].

However, while our course format uses dual training with video units, followed by guided hands-on training sessions, with direct feedback focusing on the psychomotor challenges of the technique, for La Casse et al., the hands-on session was not part of the instructional unit, instead serving only to assess the obtained skills after completion of the online modules [23].

Due to a general trend towards conserving human resources due in part to their steadily increasing expense, the course presented here needs to be compared with other efforts in the future. In addition, it is necessary to compare the extent of different courses, as students in the future need complete head and neck ultrasound training. Previous studies supported not only the value of ultrasound courses [24,25,26,27]—as supported by this very study—but also indicated the need for significant human resources. Therefore, one major focus was the incorporation of modern media to surmount attendance constraints that arose during the COVID-19 pandemic, but which might very well make a reappearance in the future. The integration of video-assisted lessons in undergraduate ultrasound education has already been explored for skills related to the collection of cardiopulmonary, gastrointestinal, genitourinary, and musculoskeletal findings [28] and POCUS skills [29]. The acceptance and subjective learning success of the videos have been evaluated positively. Nevertheless, in the case of a video-based course, additional tutoring is regarded as necessary [30]. The work of Kameda et al. demonstrated further the feasibility of video-based self-learning followed by telepresence instruction on focused cardiac ultrasound (FOCUS) for medical students. In a flipped-classroom design, participants in a FOCUS course were first provided with video instruction on the standardized examination technique and self-training. Additionally, a course via telepresence instruction was provided by a principal investigator using a video-conferencing tool. Effectiveness was evaluated using skill pretest and posttest scores [31]. Similar to our study, significantly higher post-test score values were shown after the video lecture. This supports the hypothesis that video-based self-learning followed by telepresence instruction of course participants can be an effective format for teaching ultrasound skills. Learning is greatly stimulated by this format. Active self-learning incorporating modern technology is suited to address current and future students’ needs. This work might very well represent a cornerstone towards unveiling modern and innovative forms of instruction and education, irrespective of its origin during the COVID-19 pandemic.

Limitations of this study:

Several contextual factors need to be taken into account when interpreting our study outcomes. To put our findings into context, it is important to note that the comparison between our video-based telepresence approach and other formats—specifically, the online-based course and the in-person format—draws on previous studies. These earlier studies were conducted during the challenging circumstances of the COVID-19 pandemic, which necessitated smaller group sizes due to access and hygiene constraints.

The present study was motivated by the resource-intensive nature and time constraints identified in our earlier work [21]. Our primary goal was to develop a more efficient course format that would impart the same knowledge and skill gains as the other instructional formats while conserving time and human resources.

In presenting the results of the video-based telepresence course format, the smaller cohorts from previous studies will be considered as benchmarks only. In the future, it is important to expand the participant pool, taking into consideration various factors. This comprehensive approach will provide a deeper understanding of how different instructional formats impact learning outcomes. By doing so, we can better assess the effectiveness of the hybrid course for a wider range of students.

The present study included a limited cohort size of 35 voluntary medical students. While our findings offer valuable initial insights, future studies should aim to involve larger and more diverse student populations to enhance the generalizability and robustness of results. This would allow for a more comprehensive assessment of the intervention’s impact across a wider range of learners. Another limitation is the variation in the student population. Our participant pool comprised a specific subset of students, which might limit the transferability of our findings to a broader student population. Future research could explore the effectiveness of the telepresence-guided approach among students with varying levels of prior ultrasound experience. Stratified sampling or recruitment across different levels of education could help elucidate how the intervention impacts learners at different stages of their medical training. Furthermore, the voluntary nature of participation might have introduced self-selection bias, favoring motivated and enthusiastic students. To mitigate this potential bias, future research could employ randomized controlled trials, where participants are assigned to intervention and control groups through random allocation. This approach would enhance the internal validity of findings and provide a more accurate assessment of the intervention’s true impact. One more limitation is the short observation period. Our study’s six-month duration allowed for initial insights, but a longer observation period is necessary to comprehensively assess the intervention’s long-term effects. Subsequent studies spanning multiple academic semesters or years would enable the exploration of sustained learning outcomes and potential decay rates in ultrasound proficiency. Another limitation is the comparability of the groups. The data collected in the current study were compared with a previous study, which was based on the same evaluation criteria. Thus, although there is a certain discrepancy between the compared groups, the basic system of the survey was the same.

## 5. Conclusions

In the present study, we show that an extension of our HANUS course with instruction in telepresence to include video instruction is a promising tool in ultrasound education. Effective self-learning can be supported by video instruction. The demonstrated course format can help save resources while addressing individual student needs. The technology offers great potential to expand the range of teaching in the field of teaching practical manual skills and can be considered as a cornerstone with regard to telemedical applications.

## Figures and Tables

**Table 1:** Self-assessment questionnaire already described in [21].

Self-Assessment Questionnaire					
Age					
Sex					
Clinical Semester					
Pre-course Ultrasound Experience	YES	NO			
If YES, pre-course knowledge gained through	Courses	Literature	Internships		
Self-assessment	Locating and visualizing the organ	1 = is not possible	2 = is rather possible	3 = Is possible	4 = is confidently possible
	Floor of mouth				
	Larynx				
	Trachea				
	Thyroid gland				
	Esophagus				
	Salivary glands				
	Vessels				
	Lymph nodes				
	Pathologies				
The time allocated for the course was sufficient	0	1	2	3	4
The teaching materials provided were sufficient	0	1	2	3	4
The comments of the tutors were helpful	0	1	2	3	4
The content of the course was sufficient	0	1	2	3	4
The tasks were clearly formulated	0	1	2	3	4
The teaching materials provided were clearly structured	0	1	2	3	4
The learning content did not overwhelm me	0	1	2	3	4
The group size was comfortable	0	1	2	3	4
The explanations of the tutors were well understandable	0	1	2	3	4
My questions were answered sufficiently	0	1	2	3	4
The feedback on my skills was sufficient	0	1	2	3	4
I consider the course format suitable for knowledge transfer	0	1	2	3	4
It would be useful to extend this course to other organ systems	0	1	2	3	4

**Table 2:** External assessment score already described in [21].

Assessment Category	Score Points	
manual skills	max. 8 points	
Situs orientation	0–2	Landmarks and guiding structures are recognized: 0 = no, 1 = partially 2 = relevant structures can be named
Transducer positioning	0–2	0 = no orientation, 1 = partial orientation, 2 = complete orientation
Adjusting the focus position	0–2	0 = no focus, 1 = partial focus, 2 = focus is adjusted
Adjusting gain	0–2	0 = no gain, 1 = partial gain, 2 = gain is adjusted
interaction with the patient	max. 4 points	
Patient positioning	0–2	0 = no adjustment of patient position, 1 = partial adjustment of patient position, 2 = patient position is adjusted and properly optimized
systematics of the examination	0–2	0 = no systematics, 1 = partial systematics, 2 = good systematics
Visualization of the organs	max. 20 points	
Measuring	0–4	0–4 organs or structures are measured correctly
Demonstration	0–16	0–16 organs are displayed
Explanation of the anatomy shown	max. 18 points	
Normal findings	0–14	0–16 organs are examined
Pathologies	0–4	0–4 pathologies are assessed, if no pathology then 4 points.
Score total points	max. 50 points	

**Table 3:** Characteristics of the study population. Median, maximum, and minimum (brackets) or total number of participants.

	Online Course	In-Person Course	Video-Instruction-Based Remote Teaching
Age	24 (21–29)	24 (22–34)	23 (19–31)
Gender (female:male)	6:6	14:8	9:26
Semester	8 (6–10)	6 (5–8)	7 (4–10)
Ultrasound-knowledge/experience pre-course (courses, internships, and literature)	66.6%	45.5%	42.9%

## Data Availability

The data presented in this study are available on request from the corresponding author.
